# Outputs from a model of co-research with older care-experienced people in Sweden to advance eldercare services

**DOI:** 10.1186/s40900-024-00650-7

**Published:** 2024-11-11

**Authors:** Sarah Wallcook, Ing-Mari Dohrn, Ulla Dahlkvist, Yvonne Domeij, Kerstin Green, Gigi Isaksson, Ida Goliath

**Affiliations:** 1 Department of Neurobiology, Care Sciences and Society, Karolinska Institutet, Stockholm, Sweden; 2 Stockholm Gerontology Research Centre, Stockholm, Sweden; 3 Independent Co-researcher, Stockholm, Sweden

**Keywords:** Co-research, Co-production, Seniors, Patient and public involvement in research, User involvement, Organisational development, Practice-focussed research, Community engagement, Hard-to-reach, Innovation

## Abstract

**Background:**

Within the contemporary policy turn towards co-production and co-research in Sweden, older people, practitioners and researchers alike have expressed uncertainty about how to undertake co-research. Moreover, scepticism persists about the merits and contributions of co-research and co-production to research and service development. In this paper, we aim to describe a co-research model developed with older care-experienced lay co-researchers and explore the utility of our model’s outcomes to social care research and practice.

**Method:**

In a Participatory Action Research project, we established a team of three co-researchers by professional experience and eight lay co-researchers by lived experience who were over age 75. Our team undertook a complete study cycle from inception and funding through to knowledge exchange and dissemination. Our process lasted one year and comprised three phases: the group alone establishing collective knowledge, testing knowledge in peer interviews with acquaintances, exchanging knowledge in events with multi-sector actors. We generated and analysed data concurrently in fortnightly workshops and round-table meetings using procedures inspired by framework analysis to produce themes illustrated by vignettes.

**Findings:**

We highlight our co-research model’s utility to social research, policy and practice under three themes. Expressly, how our approach (1) reaches and engages older people who are isolated at home, (2) generates out-of-the box thinking and innovative solutions for service development and research, (3) recognizes and benefits from older people’s authentic experience and knowledge. We critically reflect upon these three themes and the conditions that lead towards or away from the adoption of key co-research principles.

**Conclusions:**

Public services and research and development units working in the interests of older people can take inspiration from our co-research model when seeking to meet the challenges brought by new reforms towards closer community involvement. Despite messiness in the process, integrating and embedding principled co-research and co-production can bring clarity and structure to the issues that matter most to older people, and draw organisations closer to the communities they serve.

**Supplementary Information:**

The online version contains supplementary material available at 10.1186/s40900-024-00650-7.

## Abstract (plain language summary)

In this paper we respond to the call for clearer descriptions of how co-research is carried out and contributes to developing eldercare services and building new knowledge. Our co-research team includes three researchers with professional experience of health and social care for older people, and eight researchers aged over 75 with lived experience of giving or receiving formal or informal care. Over one year we conducted a whole research cycle (from funding and study design, to presenting findings and planning new research) in a three-phase process: (1) the group worked alone to pool their knowledge on a topic that was important to them, (2) they tested this knowledge by interviewing other older people, and (3) they shared this knowledge with professionals in research and development units, eldercare services and wider stakeholder organisations. In undertaking and describing our co-research we found that our way of working was useful for social care research and practice in Sweden, which faces new legislative and policy reforms. Specifically, we saw that our co-research model reached and engaged with older people that services often consider hard to reach due to isolation at home. Moreover, our model gave us a deepened understanding of issues that mattered which led to out-of-the-box thinking that could contribute fresh ideas to services aiming to solve complex problems. Finally, for research to genuinely benefit from older people’s lived experience, older people must have influence at the earliest opportunity in a flexible approach that breaks intergenerational taboos and provides space for knowledge to flourish.

**Supplementary Information:**

The online version contains supplementary material available at 10.1186/s40900-024-00650-7.

## Background

Co-production of public services (i.e. planning, design, delivery, evaluation) refers to a process that takes place between service-users and professionals, rather than processes of inter- or intra-organisational collaboration [1]. The term co-research shares similar characteristics, where people who are directly affected by the research are equally and genuinely involved in a process with professional researchers that generates new knowledge e.g. to prioritise research questions, design and undertake studies, analyse, and disseminate findings [2]. The two terms overlap at various points as, for example, in co-produced service evaluation where professionals may involve service-users as co-researchers to interview other service-users about their experiences and analyse the data to identify important findings [3]. A partnership approach between older service-users, staff, and researchers in service development work is a widely recommended, but relatively new phenomena [4]. As a collective, researchers, managers, and service users have perceived that co-production offers a ‘kind revolution’. Within this revolution everyone involved should live well through the co-production process and deliver more radical work that benefits public services and performs societal good [5].

In Sweden, policies promote an active role for users in care and service development, where the new Social Services Act anticipated in 2025 is expected to strengthen the requirement for user involvement [6]. This is despite a backdrop of substantial financial cuts to care services and pressure to contain costs and achieve cost-effectiveness [7, 8]. A trend towards the politicisation of co-research has been followed in Sweden since 2009 where Pestoff first identified that changes in policy contributed to the crowding-in and crowding-out of co-production in public services [9]. In the current upsurge of interest in co-production and co-research, older people, service developers and researchers alike have been found to be sceptical about co-research and lack the systems, tools, and competence to meet user involvement requirements [10–14]. Moreover, despite some notable exceptions, co-research with older people, particularly those in more vulnerable situations, has lagged in Sweden and elsewhere [15, 16]. Uncertainty about how to involve users in i.e. co-researching and co-producing is linked to the development of a number reporting tools, e.g. Co: Create Co-production matrix, GRIPP2 [17, 18] the merits and drawbacks of which are debated [19, 20].

Evaluating the effects and tracing immediate and longer-term impacts are a critical, but seldom conducted and poorly supported aspect of co-research [15, 21, 22]. A recent scoping review found a paucity of reporting concerning how and how well co-research approaches with older people were practically applied in the research process [17]. The same paper cautions against the development of how-to guides and scoring systems for co-research that could lead to aiming for the highest possible scores, efforts which may not ultimately contribute to the purpose behind the approach. Instead, the authors urge clear reporting of the process and experiences that facilitate transfer of learning about what works, for whom, and in what circumstances. In meeting this request, our paper falls somewhere between the formats of empirical report and method article. Our method section details how we practically went about the process of co-researching. Subsequently, we share what our process contributes to research and service development with an emphasis on novelty and advancing the international conversation on involvement. Consequently, in this paper, we aim to describe a co-research model developed with older care experienced lay co-researchers and explore the utility of our model’s outcomes to social care research and practice.

## Method

### Origins and evolution of the project

This project arose from a larger Participatory Action Research (PAR) project *Co-designing collaborative prevention of deterioration of health in older adults (SAMSAS)* led by co-author IG. Within this project, stakeholders including representatives from pensioner groups, and staff from civic sector, and public health and social care organisations developed their collaboration around the prevention of health deterioration for older people through a co-production process. Early in this process in a SAMSAS reference group meeting of involved stakeholders, pensioner organisation representatives highlighted a need to gather lay older people’s perspectives on collaborative health deterioration prevention. More specifically, to ensure that the perspectives of older people affected by deteriorating health were included.

To address this need, we continued with a PAR approach and developed a model for undertaking co-research with older people (reported in detail here and appended with the GRIPP2-SF checklist) while simultaneously using this model to explore perspectives on health deterioration and prevention [23]. In exploring these perspectives, we anticipated that the older co-researchers could become involved in co-producing knowledge about, and ideas for solutions that could address, important aspects of prevention that are relevant for service development.

### Our approach - co-research in relation to participatory action research

Both PAR and co-research seek to enact principles that address the power imbalance between research, practice, and the affected public and make them equal partners to deliver social change and enhance outcomes [24–26]. Indeed, the two concepts are not necessarily separate from one another, since partners engaged in PAR may be considered co-researchers that participate in and lead an array of research tasks [27]. However, we chose to make a distinction since a critical interpretive synthesis uncovered that within PAR projects, older people were often positioned as participants rather than partners and had little influence over the process [28]. By distinguishing co-research from PAR, we indicate our intention to involve older people as equal partners in all aspects of the process i.e. formulating research questions, undertaking research and utilising findings. Rather than implementing specific methods, models or frameworks; success in co-research has been attributed to ongoing adoption of key principles. Namely: (1) a systems perspective that assumes local adaptation and non-linearity, (2) creativity and human experience at the core of development and research, (3) emphasis on the process relating to framing tasks, relationships, governance, and facilitation [29]. Our ensuing description aims to highlight how we adopted these principles in our co-research project.

### Setting

The Stockholm Gerontology Research Centre is a research and development (R&D) unit, tasked with supporting regional and municipal actors in their organisational development work through practice-focussed research. We offered our project in partnership with the prevention unit at the eldercare department in Södermalm (one of Stockholm’s city districts), which integrates initiatives at community meeting places for older people.

### Ethics

The Swedish Ethical Review Authority granted permission to develop a co-research approach with older people (Dnr 2021–04645, 2022-02744-02). Our approach was ethically concerned with shaping relationships and being attentive to the varying contributions that older lay co-researchers (LCRs) wish to make combined with ensuring that practical support needs are met [16]. As professional co-researchers (PCRs), we were aware that we needed to be alert to socially engrained protective tendencies when presented with older peoples’ apparent cognitive and physical vulnerabilities. Upholding the agency of older people meant taking actions that enhanced, rather than restricted, opportunities to co-research [30]. We assumed that potential lay co-researchers could independently give informed consent to join the project. Additionally, we paid extra attention to consent procedures based on the likelihood that people with known or unknown cognitive impairments may join. In this regard, we took written and verbal consent from LCRs and peer interviewees, and followed guidance developed for upholding the self-determination and integrity of people with cognitive impairment, e.g. use of orientation statements and reminders of freedom to withdraw from the project [31]. We accepted that during relevant phases of the project, the LCRs’ role would need to be jointly developed, clarified, and potentially revisited, with regards to research rectitude i.e. access to data, ensuring anonymity and confidentiality (if not otherwise preferred), fieldwork sensitivity [32]. Consequently, we made sure that LCRs could take decisions around the visibility of their contributions and how their work would be recognised, for example, as named presenters, authors, contributors, and so on [16].

### Co-research team

Three researchers with professional experience of designing and delivering socially driven health- and practice-based research were involved. SW and IG from project inception (January 2022), and I-MD joined the project once we had commenced data generation (September 2022). To join our team of three PCRs we aimed to recruit eight to ten LCRs initially with a view to achieving a settled group membership of six to eight LCRs. We anticipated that the older people most affected by health deterioration would be those who have lived experienced of dealing with their own care needs, or the care needs of others either informally, or via formal provision. We reviewed the literature to take account of assertions and learnings from the field in how we tailored our recruitment approach and procedures to facilitate involvement around care-related circumstances (see Table 1).

All recruitment followed our partner’s first newspaper announcement, which included a clear call out to older people age 75 + to get involved in a research project about preventing health deterioration, that support would be available and that carers were welcome. 18 people responded (gendered by name as 16 women and two men) and received detailed co-researcher information sheets about the project. The information stipulated a group format, that training and selecting research methods, analysing data, and sharing results were all part of the project. Additionally, that the length, frequency and location of the workshops would be decided based on a pre-start conversation about individual preferences and needs, and that the project duration could be up to two years but that co-researchers were free to leave earlier.

Ultimately eight people – all unknown to one another and the PCRs – joined the project and the remainder declined. These people self-identified as seven women and one man, however one LCR left the group due to ill health after one workshop. This left a group of seven women aged 78–93 to continue seven fortnightly workshops in the Autumn of 2022, one of which was cancelled due to exceptional weather. In the new year, one LCR decided not to continue for unknown reasons and a protracted period of ill health beset two LCRs, one of whom later sadly died. This meant that the group ultimately became four LCRs – YD, GI, KG, UD – who continued over seven workshops with peer-interviews between January and June 2023, and seven knowledge exchange and dissemination events between May and December 2023.

Demographic information about the LCRs is presented in varying degrees due to the differences in how long LCRs were engaged in the project and what they thought was relevant and had the opportunity to share about themselves in that time. Furthermore, as our co-research progressed, LCRs reflected upon positionality and subjectivity in knowledge generation which meant that the amount and type of information they considered relevant to share generally increased over time. Additionally, the PCRs grappled with the potential injustice of presenting personal information about LCRs without presenting similarly personal information about themselves regardless of the apparent relevance of this information to the topic. Consequently, in Table 1 we present demographic details about ourselves stratified by PCRs, LCRs who completed the project, and LCRs who left the project.

**Table 1:** Demographic descriptions of the co-research group stratified by PCRs, LCRs who completed the project, and LCRs who left the project

3 PCRs	Three middle class women aged mid-forties to sixty who all visibly pass as Swedish^*^, one of whom speaks Swedish as an additional language. No disabilities or health conditions, good or very good self-reported health and participating in society in the way they want to, or in one case, not as much as they would like. All are co-habiting and have someone who would support them if they needed it.
4 LCRs	Four women aged late seventies to mid-eighties who visibly pass as Swedish^*^, one of whom speaks Swedish as an additional language. Two middle class, one who described herself as ‘underclass’ who grew up in children’s homes and experienced homelessness for more than a decade, and one who said that as humans ‘we have no class’. Two had a university or college education of three or more years, one had a shorter higher education, and one completed secondary education. Work backgrounds included social worker, psychotherapist, care assistant, author, Swedish language teacher to refugees, telephone operator, public services official. Informal care experiences for family and friends with varying intensity of involvement from occasional help to providing live-in care for more than a decade. Two self-reported very good health, two reported good or fair health together with arthritis, hearing impairment and hypertension. One used a walker and writing interpretation service, one used a home care service and one had received a positive decision for home care but found the service too expensive to use. Three lived alone and one reported having no one to help them if they needed it.
4 LCRs who left the project	Three women and one man aged late seventies to mid-nineties who all visibly pass as Swedish^*^. Work backgrounds included journalist, tax officer, psychologist, driver, teacher. One used a walker and interpretation services, one used a home care service, and one needed a home care service but did not have it. One had recovered from a serious health condition, two had joint or back pain.

* we use this phrase to reflect contemporary Sweden’s rejection of race as relevant social category in issues of injustice and inequality while simultaneously acknowledging that our experiences of racialisation do not lead to discrimination. See e.g [33].

### Co-research procedure

Our approach to procedures was based on our insights from critical gerontology and several key assertions and learnings from the literature regarding involving older people in research (see Table 2). In applying this learning, we planned for a three-phase workshop-based process over one year that would facilitate progression in group confidence and readiness to become involved in cross-sector, multi-actor collaborative exchanges.

**Table 2:** Planning to develop co-research procedures that take account of assertions and learning from the field (a selection of considerations)

Assertion/learning to take into account	Our planning response and action
Digital exclusion overlaps with health inequities so that digital recruitment and involvement approaches are more likely to exclude older people from disadvantaged socioeconomic backgrounds, or with disabilities and health conditions [34]. Moreover, the Covid-19 pandemic may have confined and exacerbated isolation among digitally excluded older people in Sweden whose social interactions may have become more infrequent or ceased through voluntarily following age-group targeted public health guidelines [35, 36].	Open call for co-researchers aged 75 + via the local newspaper delivered for free to all households in the neighbourhood with widest readership among people over 65 years old. Posters at meeting places for older people. Information exchanged via post, phone calls, email, text messages according to personal preference. Heeded preference for physical, not digital, meet-ups as public health guidelines had eased. Recruitment information stated that support to participate would be provided to people with physical, memory and cognitive impairments and welcomed significant others.
Equal status and power sharing are facilitated by monetary and non-monetary recognition. However, the professionalisation of involvement may be more attractive to people with greater experience of research and higher self-belief that they have something to offer, exacerbating inequalities and limiting the potential to include valuable, marginalised voices [37, 38]. Furthermore, complex administration in relation to payments and taxation arrangements can create new problems and risks for people that become involved in research [10].	Voluntary model with a focus on non-monetary reward (refreshments, celebration events, visibility at all stages, expenses covered) due to financial constraints. After six workshops, the project received funding and the co-researchers also received a fee per activity where contracts and invoicing were adapted and simplified to facilitate ease of receiving payments.
Creating inclusive involvement opportunities means planning to do less over a longer period and adapting methods, tasks, and activities to accommodate cognitive, physical, sensory impairments, fatigue, and language barriers. Events taking place beyond the normal meeting structure may require extra coordination support for practical arrangements [32, 39].	Selecting an accessible location in the heart of the local community with short walking distances close to public transport links. Providing support to participate i.e. writing interpreters*, large print, information in multiple formats, breaks, refreshments, training, welcoming supporters and/or cared-for persons, booking travel and travelling alongside. Calling/messaging reminders ahead of meetings. Being available for contact between meetings to receive and discuss ideas, reflections triggered by e.g. TV programmes, news articles, books related to the topic.
Co-researchers’ health and life circumstances may change and lead to them ending their role in the project. However, tokenism may arise in instances where co-researchers’ have limited scope and opportunity to influence practice and see their results recognised and utilised due to too short timescales [27].	Planned a full research cycle in pilot over one year to include dissemination and knowledge exchange activities for those who could be part of the whole project. We aimed for flexibility and valued all contributions equally, respecting that LCRs would decide on their own level of involvement [40]. Consequently, no aspect of the project was mandatory, and each workshop activity was designed to deliver freestanding outcomes to each co-researcher as well as our inquiry that would also inform planning for the next workshop.

*The writing interpreters were organised by an LCR who was a registered user of, and advocate for that regionally funded accessibility service

### Data generation

Enacting the principles underpinning our approach meant being agile with planned workshop activities and reflecting in action to respond and adapt relationally to the LCRs. This was particularly the case in the first phase of getting to know one another where people’s interests and capabilities needed close attention and the pace of working together was uncertain. From the get-go, activities were designed with a two-fold intention, firstly for their own potential to gather relevant information, and secondly to generate curiosity and questions used to inform future planning.

#### Phase 1: establishing the LCRs’ collective knowledge – the co-research group alone

In this phase, PCRs left the focus of the future inquiry into health deterioration prevention deliberately open to gauge the LCRs level and direction of interest in the topic and allow scope for them to co-produce a burning question that they wished to answer. Here, the PCRs asked a lot of questions and listened holding back their own knowledge of the research field or personal perspectives [41]. Since the LCRs had lived experience that contributed to their question, the workshop format in this phase was used to promote community building and pooling of LCRs expertise and skills [42]. This pooling and shared learning about one another also contributed to forming and establishing roles within the project. LCRs did not need to prepare for workshops, but occasionally set themselves tasks for intervening weeks or continued their own inquiry through i.e. books, film, media that they would share with the co-research team and that contributed to the enquiry and future directions of the project.

Summary Table 3 gives an overview of each workshop, its activities and the new questions that arose and influenced the aim and activities for the subsequent workshop. PCRs designed the workshop activities as individual and joint exercises that stimulated discussion and reflection around the question and provided a structure in which LCRs could share their own stories and lived experiences. These activities were grounded in a basic improvisation principle of meeting all contributions positively to accept and build upon the stories to establish an accepting space and avoid negative blocking [43]. LCRs produced written and visual records through the activities, from which SW produced written record that was given back to and agreed by the group at the following workshop. All workshops in phases one and two were audio recorded.

**Table 3:** Workshops phase 1. Iteratively developed workshop aims, procedures, and unanswered questions

Theme	Aims, procedures, unanswered questions
Experiences of the topic	Aims: To learn about one another and why the topic is personally meaningful Procedures: Group discussion through commonly known games (Bingo, Snakes and Ladders) Question: What are the role expectations?
Shaping roles	Aims: To share the detailed project background, and map role expectations and contributions Procedures: Presentation, group discussion, analytical sorting exercise Question: Can we change the angle on the topic, and terminology?
Preliminary topic framework	Aims: To present and re-sort collated data on the new topic into preliminary categories Procedures: Dynamic visual mapping on a large surface with group discussion Question: Is this a focus group method that we are using? Can we try an individual approach?
Write a diary	Aims: To capture individual accounts of the topic Procedures: Home-based diary task on one specific day Question: How did others experience this task?
Exploring diary method	Aims: To examine and reflect on other experiences captured in the diary method home task Procedures: Storyteller’s chair/talking stick, situating and reflective discussion Question: What differences do these diaries make to our framework?
Prioritising within the framework	Aims: To refine and prioritise within the framework based on diary data Procedures: Group sorting exercise and individual voting cards Question: How can we know that others would agree with our framework and prioritisation?

#### Phase 2: reaching out more widely to test knowledge – data collection with ten peers

The trigger for entering phase 2 was the final question arising in phase 1 (see Table 3). This question confirmed that the LCRs had reached the limit of their internal group understanding of the phenomena and now sought to confirm or disconfirm the knowledge they had generated by exploring new perspectives [44]. Consequently, this phase saw the LCRs taking an active role in designing and undertaking a study that attended to the focus they had identified and involved them recruiting from a wider pool of older people and generating new data. Activities therefore took place outside the workshop format, since as a first step, LCRs tested the water in everyday conversations within their own networks of acquaintances (see Table 4). In these conversations, LCRs talked about the study, gauged interest in participating and inquired about preferred conditions for participating (i.e. writing or talking, being interviewed by their acquainted LCR or by a LCR unknown to them, in their own home or another place). From this preliminary step, LCRs returned to the workshops to develop a recruitment strategy and data gathering procedures [23].

Working from a first draft produced by the PCRs, the LCRs then revised and finalised an in-depth conversation guide, a logbook guide, and a sociodemographic questionnaire. They received training in peer-interviewing through teaching and role play and received a tips sheet for use in the field derived from a previous project [45, 46] that was revised to incorporate co-researchers’ reflections on the training. LCRs then considered their individual readiness for interviewing and planned what role and degree of leadership they and the PCRs respectively would take. Ultimately, this meant that one of the PCRs (SW) attended to the practicalities of coordinating the scheduling, equipment, and administrative requirements (i.e. travel, venue, forms, recording devices) so that LCRs could focus solely on interviewing. The interviews were conducted according to LCR’s and participants’ preferences meaning that two LCRs interviewed with support from PCRs, two interviewed alone, and only one LCR interviewed their acquaintances while the other three interviewed people unknown to them. The interview guide included a set of questions for LCRs to reflect upon post-interview either in an audio recording, writing down their thoughts, or having a recorded discussion with a PCR. Ultimately, the LCRs conducted ten peer interviews, which were audio-recorded, and received one logbook. Further workshops in this phase are described in the analysis sub-section.

**Table 4:** Workshops phase 2. Iteratively developed workshop aims, procedures, and unanswered questions

Theme	Aims, procedures, unanswered questions
Planning to test the framework	Aims: To identify recruitment and data collection opportunities Procedure: Group discussion and planning for informal conversations with acquaintances Question: What data collection approach suits prospective participants’ preferences?
*Holding informal conversations*	*Aims: To gauge acquaintances’ interest in participating in the project* *Procedure: Informally chatting about the project and asking questions* *Question: What if no one I know wants to take part?*
Matching method to participants	Aims: To collate and take account of informal conversations in selecting an approach Procedure: Sharing anonymized information, formal plan for data collection and recruitment Question: What questions should we ask to access participants’ experiences?
Designing an interview and logbook guide	Aim: To compile questions and content for data collection guides and invitation letter Procedure: Roundtable to gather, discuss and alter formulations written up in large format Question: How do we interview rather than converse?
Practicing interview techniques	Aim: To learn about and practice interviewing and decide recruitment/data collection roles Procedure: Training in interviewing, interview in pairs, group feedback, agree final guide Question: No question – send out information and book interviews
*Conducting interviews*	*Aim: To generate data that tests the framework with participants* *Procedure: Conducting audio-recorded interviews in a place chosen by participants* *Question: What is happening in the other interviews?*
Mid-interview phase reflection	Aim: To share new knowledge arising from and experiences/challenges of interviewing Procedure: Talking stick, situating and reflective discussion of newness, difference/sameness Question: What happens next to these interviews?
Post-interview framework analysis	Aim: To analyse the interview data and refine the framework Procedure: Sorting segments of data in relation to the framework’s priority areas Question: How do we present our work?
Constructing vignettes	Aim: To condense our findings into interpretable vignettes Procedure: Group discussion and revision of drafted examples Question: How can we make a difference with this work, who needs to know?

Rows with italic text indicate activities taking place outside the workshop format

#### Phase 3: dissemination and knowledge exchange with wider stakeholders

Dissemination began at the outset of the project with a focus on regular dialogue between PCRs at the Stockholm Gerontology Research Centre, Eldercare at Södermalm city district, and partners in the wider parent project, SAMSAS. This dialogue took the form of regular updates via meetings and email contact between SW and stakeholders, who commented on how the co-research project progress related to their own organisational interests and priorities. The LCRs knew about these updates and received confirmatory feedback that stakeholders were interested in their work and arising priorities. This behind-the-scenes dialogue anchored awareness in the group that the channels were in place for the co-research to influence policy and practice [27]. However, care was taken to not enter the LCRs directly into a premature dialogue and instead leave them free to focus on their own prioritisations without undue pressure from other agendas. As our co-research consolidated into clear, well-anchored findings, updates progressed to knowledge exchange events with stakeholders and interested audiences from research policy and practice [47]. These events highlighted the implications of our work seen through the eyes of stakeholders who asked questions and shared reflections that illuminated which aspects of the findings they considered most important and meaningful. Our knowledge exchange activities broke with the workshop format and took a variety of forms described in Table 5. For each event, the LCRs attended a preceding planning meeting to prepare the presentations and programs/agendas with PCRs and shared their reflections after the event. The LCRs role in the events quickly developed from discussing the findings presented by PCRs, to presenting the work directly to their audiences with the PCRs. Planning meetings and reflections were audio recorded, whereas excepting the two roundtable meetings knowledge exchange events were not audio recorded.

**Table 5:** Phase 3 knowledge exchange and dissemination activities, description of context, roles, and outputs

Type of event	Context, roles, output
Seminar	Invited to present and discuss the project with a partner R&D organization SW presented, KG and UD discussed We gained insight into our work’s relevance to R&D, our achievements and progress
Roundtable meeting part 1	Presenting the project to Södermalm city district partners who shared spontaneous reactions IG chaired, SW presented, KG, UD, GI, YD discussed in a popcorn style dialogue* with partners Immediate respect for the value of the findings to eldercare which demanded consideration
Roundtable meeting part 2	Hearing about Södermalm’s current working practices, plans and ideas arising from meeting 1 IG chaired, Södermalm partners presented, SW, KG, UD, DI, YD discussed popcorn-style Baton passed^§^ and ideas generated based on shared interests and partner’s needs
Supporting a successful research bid	Hearing how ideas in generated in meeting 2 were drafted into a funding application SW shared a lay description of research, KG, UD, DI, YD gave feedback, refined e.g. the title Communicating to funders the significance of the project to LCRs through a support letter
Seminar	Gave a seminar within the Stockholm Gerontology Research Centre’s own seminar series SW, UD, GI, IM-D, KG, YD presented and discussed audience questions Creating a dialogue with wider stakeholders i.e. attending politicians, strategists, practitioners
Conference presentation	Presented at the national Welfare Research and Development conference SW, IM-D, UD, GI, KG, YD networked, presented, and discussed questions with attendants Made an equal contribution to the field with delegates from across the country
Lecture	Invited to give a lecture for a grass roots organisation SW, UD, GI, KG, YD presented to practitioners and older peers interested in health care Peers agreed with our message and the organisation planned to make immediate changes

* in a popcorn dialogue, reflections, sharing and discussion are freely triggered through curious open questions [48]

^§^ by baton passing, we mean that co-researchers entrusted their findings to partners to take forward

### Analysis

Consistent with PAR, analysis took place within the group workshops where our discussions and interactive activities, aside from generating data, were also analytical through our questions and reflections [49]. During these activities, LCRs generated written notes of their discussion, reflections, and experiences that they sorted and categorised. These categories guided the selection of rich and relevant workshop segments for verbatim transcription by a third party contracted to provide a confidential and secure service. Additionally, SW listened repeatedly to workshop audio recordings, took further notes, wrote reflexive journals, and had analytic discussions with I-MD and IG between each work workshop. As the project progressed, analytic exchanges also took place with LCRs, who called or emailed SW to discuss new sources they had found, things that had stuck with them from the workshop, or revisions they wished to make to previous ideas and contributions. The outcomes of this analytic process from and between each workshop were then presented back and discussed at the next workshop, which served to increase the whole team’s familiarity with the data and the directions being taken. These summaries included the questions asked, categories that had been produced, data underlying each category and the outputs that we had arrived at.

In phase one, analysis was open, following the data and categories and we took inspiration from the procedures used in framework analysis to inductively produce themes [50]. In phase two, analysis moved towards deepening our insights by confirming or disconfirming the themes we had constructed [44]. To establish our themes, rather than LCRs reading complete transcripts, SW produced descriptions in the form of vignettes as a way to condense large amounts of rich data into manageable chunks. Vignettes are short stories that encapsulate the complexities and sensitivities of individual and group experiences and can be used as a tool for analytic dialogue to clarify judgements about the norms and values conveyed [51]. This was a technique that SW had previously developed and found to be inclusive and effective for communicating and discussing preliminary findings in analytic consultations with people with mild stage dementia [52]. These vignettes were often sufficient to prompt discussion about whether any important aspects from the interview had been missed and whether this condensing process had produced an accurate vignette that matched the LCR’s recollection of the interview. Having developed draft vignettes in phase one, these drafts were then sequentially refined by both LCRs and PCRs by combining in the vignettes from phase two. Phase three, contributed further to this deepening and refining by offering discussion and reflection that helped situate our work within the sociopolitical context that shapes eldercare practices. We refer to interactive and participatory activities within this phase as Knowledge Exchange as they formed a dynamic and iterative process which brought us together with our stakeholder partners in eldercare to exchange ideas, evidence, and expertise [47]. These activities helped us refine how we articulated the findings in the vignettes, honed our introduction, key messages and discussion points, and sharpened our perception of which audiences would best utilise which aspects of our work. Consequently, our findings feature these vignettes which are used to illustrate the data underpinning each outcome.

## Three findings and contributions

In reflecting upon our co-research process, we highlight three themes useful to social research, policy and practice. Expressly, we detail how our approach: (1) reaches and engages older people who are isolated at home, (2) generates out-of-the box thinking and innovative solutions that cross institutionalised boundaries, (3) recognizes and benefits from older people’s authentic experience and knowledge. We critically reflect upon how these three themes relate to the adoption of key co-research principles. Namely: (a) a systems perspective that assumes local adaptation and non-linearity, (b) creativity and human experience at the core of development and research, (c) emphasis on the process relating to framing tasks, relationships, governance, and facilitation [29].

### Reaching and engaging older people who are isolated at home

We learned that peer-interviewing can be a powerful outreach tool, particularly when the interviewer has access to older people who they know have a lot to contribute, but would typically be regarded as ‘hard-to-reach’ (or seldom heard). By ‘hard-to-reach’ in this specific context, we mean people that would ultimately like to talk about their experiences, but for myriad reasons would not or could not go outside home or have (more) strangers coming in.

LCRs who had a network of friends and acquaintances that included seldom heard people broached participating in an interview in a personal, unhurried, and gentle way. Since they knew both the person and the project, they were able to boost self-belief in the value of participation by pointing out specific examples from the person’s life that the research would benefit from hearing about. Quite often, LCRs already had these people and what they knew of their experiences in mind when they were arriving at the first frame for wellbeing (i.e. before the decision to conduct peer-interviews) and designing the study (i.e. planning to conduct peer-interviews).

In some instances, the LCR had already begun talking about their own involvement in this project with their friends/acquaintances. This meant that the question of participating did not need to be broached at the first instance, and the topic of the project, and eventually possibly participating in it, was a secondary conversation piece, simply tucked into an otherwise social or helping occasion at home. So, the idea of conducting peer-interviews and becoming an interview participant was more a seed that germinated over time in dialogue between LCRs and their network. For some friends and acquaintances, this seed germinated no further, but for others it grew to formalized participation in a research interview. Building further on the comfortable and organic nature of this recruitment approach meant continuing the conversational format to consider “how would you like the interview to be?” and giving examples.

It is important to point out that the process of recruiting and engaging participants was highly varied between LCRs and so was the nature of their relationships with participants. Most interviewees preferred to be interviewed by a LCR who was unknown to them and preserve the boundaries with their co-researching friend/acquaintance. But two participants preferred to be interviewed with their LCR present and this LCR in turn preferred to also be supported by a PCR. We give an example of her role below:

*One LCR had been a carer all her working life and her relationship with the participant incorporated that caring and assistance role*,* i.e. visiting her in hospital or at home and doing any everyday tasks that needed to be done. On the day of the interview*,* without doing anything extraordinary in their relationship*,* this LCR gently supported the interviewee to be the host in their own home and take charge. For example*,* she poured refreshments into the glassware the interviewee had chosen*,* and brought them to seating places as directed by the interviewee. As the interview with the PCR progressed*,* the LCR would occasionally interject saying “I’m thinking of the time you told me about…” to add or prompt for more details to a specific example the interviewee was giving. And at the behest of the interviewee and with ease and familiarity with the environment*,* she could go and get an object from another room that the interviewee wished to show. The LCR remained behind after the interview to wash up and put and away*,* and help with other tasks unrelated to the interview before leaving.*

In our exploration of this finding and through the example given we highlight our co-learning about the interplay between relevant factors that we perceive contributed towards ameliorating barriers to engaging specifically ‘hard-to-reach’ older people. Namely: *care* in the LCR/interviewee relationship, *time* to let the idea of participating develop organically, and *familiarity* with the interviewee’s home environment and personal match to the topic, which together empowered the person to *host the interview in their own home*.

### Generating out-of-the box thinking and innovative solutions for service development and research

Taking a salutogenic perspective on the topic meant that LCRs and peer-interviewees naturally came with solutions that accompanied their issues in the spirit of “if this helps me, it may help others”. Often these issues arising at a personal level could be solved by a simple exchange of information between LCRs. However, in some instances that information used by another person had a different outcome so that instead of solving the issue a new related problem arose that required its own solution. We give an example of this below:

*One LCR organised writing interpreters to come to the group for her own accessibility needs which in turn benefitted another LCR’s hearing impairment who had no prior knowledge of this funded service. This second LCR signed up for the service. However*,* when attempting to book and use the service as a group member*,* the service decided that our activities did not qualify for receiving funded writing interpreters and provision was stopped.*

Exchanges such as the example above served to further articulate and deepen our understanding of the issues we were highlighting and their consequences, as our questions became “what do some people know, and others not, and why?”, and “what works for some people, but not for others, and why?”.

We noticed that our process of locking down together to build the group dynamic and establish the legitimacy of using one’s own experiences as a source of knowledge generation strengthened a sense of safety in the group. This safety within the group dynamic provided the freedom to hypothesise about solutions and make suggestions at a societal and systemic level that one may otherwise feel misplaced to comment on. The freedom to think accrued along with the gestation of issues over time, as issues were re-encountered in later discussions and in the exploration of peer-interviews. Patterns and commonalities therefore became more perceptible allowing us to articulate new angles and perspectives on an issue. These new perspectives sparked alternative ideas and enabled new community-based possibilities for how, and crucially, with whom locally relevant, simple, and potentially cost-effective solutions could be generated to improve and enhance older people’s wellbeing. We give an example of this below:

*One LCR shared her experience of using the disability travel service which meant*,* among other things*,* dealing with long waits in uncomfortable conditions (exposed to the weather*,* no seating). Another LCR responded that perhaps the service could provide time-to-destination updates for users. Several weeks passed*,* and a third LCR discussed a similar example from one of her peer-interviews adding that it is not a safe situation to be in. A fourth LCR wondered aloud whether the housing associations for apartment blocks could provide fold-down seating in the lobby for people who needed to wait.*

Our example highlights the framing “I wonder if…”, which we believe contributed to keeping our solutions and suggestions curious, relaxed, and unburdened by expectation, which in turn opened the space to proffer alternatives without criticism or judgement to the first idea. By beginning with co-researchers’ and their peer-interviewees’ priorities and concerns, we noticed that the solutions generated were free from institutionally imposed limitations and disciplinary boundaries. Consequently, we implicated new societal actors, in this case, housing associations. This meant that rather than starting in a multi-actor collaboration that includes older people, our co-research process highlighted that older LCRs over time identify new multi-sector actors that they consider beneficial to engage in next steps. Such next steps would involve LCRs establishing new partnerships and collaborations that include these actors to tackle the issues identified that threaten older people’s wellbeing potentially using the generated solutions as starting points.

In summary, although we decided early on that our focus would be on articulating issues and framing them as priorities that impact wellbeing rather than thinking “how could these issues be solved?”, we found that drawing a hard boundary that disallowed consideration of solutions was undesirable. Instead, we found that an important part of our process was proffering, recording, and parking solutions and suggestions for future consideration and investigation. The outcome of including possible solutions in our process was twofold. Firstly, that the complexity of issues could be articulated more completely by exploring their solvability. Secondly, the untapped potential of our project design was highlighted since we realised that a unique dimension of our co-research approach was the generation of innovative solutions for which we had no scope to take forward. We argue therefore, that the co-research approach itself can form a solution for articulating and, if sufficient scope is built into an enquiry, addressing problems as they arise in a dynamic society. And further, that by going outside the scope of a service’s normative focus and influence, the solutions generated have the potential to overcome the confines of institutionalised thinking and divided responsibilities between different agencies.

### Recognizing and benefiting from older people’s authentic experience and knowledge

Having begun in a voluntary capacity, once our group received funding to pay co-researchers, we discussed the impact of these payments in terms of being a facilitator or barrier to research involvement. One member of the team often referred to herself as ‘not as good as’ the others in the group for example, ‘I’m not as good with words as the rest of you’. She nodded strongly in agreement when we discussed the likelihood that people with lower self-belief would think they were not worthy of being paid, and therefore not become involved. People who feel hindered in comparison to others and perceive others as more intelligent, more capable of communicating and reflecting, with wider knowledge and greater past professionalism, may be less likely to feel deserving of payment. We argue that, while greater self-belief is an oft-cited consequence of being a co-researcher, greater attention in the planning phase is warranted to mitigate high self-belief acting as a prerequisite for becoming a co-researcher. In this regard, voluntary models can be useful for facilitating involvement at the outset, and can make it more acceptable to derive personal and social benefits without co-research becoming a job.

Our partners in the department of social care were crucial for reflecting to us the role that older people as co-researchers played in knowledge generation. They were unequivocal that our process of involving older people at the earliest possible opportunity to get their perspectives on which topics needed to be defined, rather than getting their perspectives on a pre-defined topic was a key learning and inspiration for organisational development. We reflected that without the co-researchers’ initiative and mandate there would have been little impetus to shift the enquiry from the dominant policy discourse of prevention of health deterioration to promotion of wellbeing. Moreover, having this early influence increased the co-researchers’ stake in the enquiry and acted as the catalyst for accessing their experience and knowledge since they were then able to focus on what really mattered to themselves and other older people.

*Having heard our results presentation*,* our partners returned with their reflections on our process and ideas for what needed to happen next. They described our process as providing a much-needed tool that could genuinely take account of and incorporate older people’s experiences and competence in social services’ development across a broad range of issues. They contrasted this new*,* engaging process with the current approaches they were using*,* which they reflected yielded less richness and nuance*,* and provided belated and restricted opportunities to utilise older inhabitants’ perspectives. However*,* they highlighted that they lacked models and tools within the organisation to implement and facilitate such a process. Having identified a need for capacity building and continued support from the PCRs and the Stockholm Gerontology Research Centre*,* our partners and the LCRs*,* supported a larger-scale successful funding application which would tackle these needs.*

We regard having early influence to build the enquiry around what really matters capitalises upon a heightened sense of urgency which together comprise prerequisites for the first two findings. Being secure that the topic matters form the necessary bedrock for reaching out to other older people and engaging them in the same question. And with older LCRs as the keys to accessing the experiences of more, and more isolated older people’s perspectives, an authenticity is gained in the knowledge being produced. By keeping the interpretive power more firmly located within a group of older care-experienced people, we found that the core of what matters was more impervious to external driving forces that may otherwise have influenced knowledge generation. For example, not bowing to any pressure to frame the knowledge in relation to current policy, discourses, or upcoming trends.

Furthermore, we noticed that when older LCRs conducted the interview, subjects that can be regarded as inter-generationally taboo to talk about, for example sex and dating, gained an everyday neutrality. These conversation topics flowed spontaneously and with unguarded ease requiring no specific planning, framing, or introducing to take place. This is what we mean when we talk about the authenticity of experiences that were tapped into by our co-research process. That the older LCRs and participants were able to contribute their authentic experiences with no need to package them according to the agendas of others or normative considerations of e.g. social acceptability.

Consequently, we also reflected that our co-research process offered a dynamic and active way to take account of and utilise older people’s knowledge and experience which also builds a sense of community.

## Discussion

In this paper, we have responded to the call for more detailed descriptions of the process of conducting co-research [17]. We describe how our three-phase approach covered all aspects of the research cycle. Specifically, how we generated our research question (phase 1), designed and undertook a study (phase 2), exchanged knowledge and applied for further funding (phase 3), and how data was generated and analysed in all three phases. Furthermore, we reported the contributions our co-research process made to practice-focussed research and service development in terms of reaching seldom heard older people, generating innovative solutions, and genuinely benefitting from the target group’s authentic knowledge. We critically discuss each of these outputs in the spirit of continued reflection and reasoning about the conditions that lead towards, or away from, principled co-research [53].

Our process, which involved LCRs gathering their own data from among their peers, enabled us to hear from people who would otherwise not be heard due to isolation at home. We regard the time that LCRs were able to invest, and their sensitivity in recruiting their acquaintances as central to increasing participation and capacity within our study, an advantage which has been contended in previous research [54]. Further, we highlight that the intimate knowledge that one LCR had of her interviewees’ personal experiences is somewhat unusual and alludes to a tricky ethical balance between neutrality and bias. However, in having this knowledge, this LCR was able to gently nudge and coax open, honest, and enriched responses, and reassure the respondent that what they had to say was valuable and guarding against an extractive experience for both parties [40, 55].

Further ethical quandaries abound when innovations are generated, but there is limited scope to take those ideas forward in practice or at scale. In our process this meant managing expectations by communicating about which aspects we would focus on (i.e. exploring solutions as part of problem definition) at which other aspects’ expense (e.g. co-producing and implementing solutions). And further, appraising and acknowledging the limits of our likely influence at a wider scale [53]. Having an ethical exit plan and consent from LCRs to pass the baton over to our partnership organisation was a cornerstone in upholding PAR’s principle of taking action and offering a platform to create lasting and meaningful change [27]. However, our exit plan has meant multiple goodbyes, before coming together again in new dissemination activities (i.e. writing this and other articles), to the point where we have not truly ‘exited’ at all. Indeed, our relationships to one another have, to some extent, professionalised, since PCRs now have a greater awareness of LCRs’ unique competences and when it is relevant to call upon that competence in new streams of work. Additionally, since our co-research sowed the seeds of continuation for a new programme of research that has been granted funding, we anticipate that the LCRs experience and expertise may be called upon again. This would be prudent given the extensive contribution that LCRs have made to SRGC and vice-versa and the stake they have in having influenced the new research topic. However, continued involvement of the same LCRs in any research project also poses a risk that prior experience of co-research becomes prioritised to the detriment of inclusive involvement, while lived experience could become more tenuous in relation to the topic [37]. We consider therefore, that even the protracted nature of our goodbye with our continued reflection upon the purpose and appropriateness of involvement has been vital for our process of knowing and for contributing to the ‘messiness’ of PAR [56]. Moreover, we acknowledge that although our process appears neat, structured and logical in this article, the reality (in concert with other PAR studies) was situationally and relationally dynamic and required constant ethical attention to co-research and PAR principles [57, 58]. In this dynamism, roles were fluid, changing over time in terms of the positions LCRs and PCRs occupied and the degree of leadership they were comfortable to assume. Consequently, defining roles and distributing leadership between PCRs and LCRs was situational and more often roles and leadership were continually renegotiated, and decisions were taken together, democratically as a group.

Indicators used to demonstrate that co-research principles have been successfully upheld include the positive personal outcomes to LCRs - such as feeling valued and heard, increased self-confidence and self-worth, developing new skills and making a difference [16, 25]. These benefits to co-researchers have naturally led to the question of whether to provide co-research processes as an intervention to older people [59], since being involved in research can be socially enriching and become a meaningful part one’s life [52]. However, here we highlight that the prime purpose of co-research is to generate novel and relevant knowledge that is collectively needed and benefits society at large. Serving the collective cause is crucial for reaping the personal rewards of co-produced knowledge. Conversely, these rewards become costs when only personal or localized advantages are perceived, and these costs are intensified among people with low self-efficacy [60–62]. To create an intervention of co-research is to subvert its purpose and put the achievement of personal benefits in focus. This would entail a patriarchal turn that radically alters the way in which co-research is undertaken with inherent risks of negative ramifications to empowerment [58].

When evaluating the ‘success’ of co-research, we argue that it is the applicability of LCRs’ lived experience and knowledge to the research, and whether their time and contributions have been equally valued that ought to be a central consideration [63]. However, we are aware that our process may be subject to common criticism that so much resource is spent on such a small group of LCRs who cannot claim to represent the views of older people more generally. This is a paradigmatic mismatch that pervades not only academia but also the general public’s perception of what is valid and reliably produced knowledge. “We are only we, how do we know we can rely on our own knowledge?” was the question internalised by the LCRs and that arises time and again among professionals and colleagues ascribing to a positivist, evidence-based paradigm that seeks objective truths [64]. Despite the long history and contributions of participatory research methods to the scientific and practice fields, it can still be a challenge within academia and the general public to achieve recognition that the LCRs’ lived expertise is a valid source of knowledge [65]. Contrarily, such challenges were not encountered among our partners in policy and practice, who regarded our process as a strategic and systematic way to include a variety of perspectives. Moreover, while practitioners can experience policy tensions that divorce practice from older people’s realities, hearing lay perspectives offers a familiarity that leads to intuitive knowledge that confirms and expands their own practice-based experiences. Within the current health and social care policy landscape in Sweden, there are two parallel trends vying for status and influence in organisational development. On the one hand, data-driven knowledge with a heavy emphasis on systematic and statistical follow-up, and on the other hand, co-production and user involvement [6, 66]. The tension between these two trends has been noted before as with Pestoff (2009) who posited that co-production processes are particularly vulnerable to being crowded in and out by public policy [9]. This crowding out becomes convenient when co-research approaches are deemed unsuccessful, despite the fact that it can be PCRs’ attitudes and skills that impact success [60]. We advocate, that these two trends should be integrated with another to better approach the wicked problems faced in practice-focussed research and organisational development contexts. However, for researchers from more positivist traditions, this entails a radical shift in thinking and a boldness to enter into the fray of participatory messiness and uncertainty with no road map.

Calls for clear descriptions of how co-research was undertaken can be regarded as an appeal for certainty and road maps. However, addressing these calls contributes to continued paradigmatic tensions since there is a lack of compatibility with the principles that explicitly mean co-research is necessarily context specific [19]. Thus, the transferability of the specifics of this model to other contexts are rightly limited and may not lead to the same outputs when applied with other people, in different settings, disciplines and epistemologies. For example, we argue that specific aspects identified as important for upholding the principles of co-research in other contexts, such as payment [37] and sending advance materials to LCRs [39], may have been detrimental to empowered involvement in our own process. Consequently, we argue that safeguarding against disempowering co-research cannot be guaranteed by uncritically carrying out a set of specific actions that were seen to achieve empowerment in another context. This means that we urge co-researchers to maintain a critical eye on burgeoning efforts to promote and standardise public and user involvement in research and service development. Even within our own future interdisciplinary study plans that embrace a variety of research methods we need to carefully consider and evaluate which aspects of our model can be applied and how our co-research approach would function. Ultimately, principled involvement should centre a contextually flexible, relationally dynamic, and critically reflective approach that enables studies to be conducted within different research traditions.

## Conclusion

Undertaking our co-research with older people in three distinct phases that established group security and confidence before involving peers and then professional stakeholders facilitated the build-up of knowledge. Within this model of working, we realised the potential of co-research to reach older people who are seldom-heard due to isolation at home and generate out-of-the-box thinking that can contribute to designing innovative solutions. Moreover, we regard our knowledge as benefitting from a certain authenticity since our co-research model facilitated access to experiences and topics that may otherwise be intergenerationally taboo to discuss.

Organisations working in service of older people and undertaking practice-focussed research can take inspiration from our three-phase co-research model to meet the challenges of new reforms towards closer community involvement. In taking this inspiration, we encourage critical and contextual reflection on the extent to which aspects described in our model can and cannot be applied in other co-research processes. Finally, we encourage a willingness to enter the fray of this paradigm shift, and a continued commitment among co-research teams to seek a balance between structuring and embracing the messiness of this predictably unpredictable practice. Despite the apparent blurriness, co-research can deliver clear, well-organised and practical benefits to eldercare services and practice-focussed research and development work.

## Electronic Supplementary Material

Below is the link to the electronic supplementary material.

Supplementary Material 1

## Data Availability

The datasets generated and analysed during the current study are not publicly available due to the risk of compromising the co-researchers’ integrity and ownership of the findings.

## Abbreviations

PAR: Participatory Action Research
SAMSAS: Co-designing collaborative prevention of deterioration of health in older adults
GRIPP2-SF: Guidance for Reporting Involvement of Patients and the Public-Short Form
R&D: Research and Development
LCR: Lay co-researcher
PCR: Professional co-researcher
