# Supplementary material for: Outputs from a model of co-research with older care-experienced people in Sweden to advance eldercare services
Source: Res Involv Engagem. 2024 Nov 11;10:121. doi: 10.1186/s40900-024-00650-7 (PMC11556169; doi:10.1186/s40900-024-00650-7)
Supplement: Supplementary file 1 — Supplementary Material 1 [file 40900_2024_650_MOESM1_ESM.pdf]

Appendix: GRIPP2 short form

For Wallcook et al. Outputs from a model of co-research with older care-experienced people in Sweden to advance eldercare services

From: GRIPP2 reporting checklists: tools to improve reporting of patient and public involvement in research

Section and topic	Item	Reported on page No
1: Aim	Report the aim of PPI in the study	3
2: Methods	Provide a clear description of the methods used for PPI in the study	5-12
3: Study results	Outcomes—Report the results of PPI in the study, including both positive and negative outcomes	12-16
4: Discussion and conclusions	Outcomes—Comment on the extent to which PPI influenced the study overall. Describe positive and negative effects	16-18
5: Reflections/critical perspective	Comment critically on the study, reflecting on the things that went well and those that did not, so others can learn from this experience	12-18

1. *PPI* patient and public involvement
